# 

**Published:** 1846-12

**Authors:** 


### American Journal and Library of Dental Science.

---

As this Journal has become the organ of the American Society of Dental Surgeons, and as its name has been somewhat modified, it may be considered as having entered upon a new period of its existence; and its former publishers congratulate themselves and the profession on its good fortune. Its perpetuity is now considered as settled. The profession will always need a periodical devoted to its interests, and who is better able to conduct it, than the only regularly organized and extensive association of dental surgeons in the world. We repeat, that we congratulate ourselves and our professional brethren on an event so auspicious to the science of dental surgery, as the formation of a national society and the transfer of the journal to that body. The work can now be regularly issued, whether its circulation be co-extensive with the profession or not. If the subscription should be limited, the issues can be made to correspond with the demand, so that the expense to the society may be kept wholly within its means.

The American Journal and Library of Dental Science, is now proposed to take an independent ground, and will hereafter solicit no favours from those members of the profession who are opposed to the dissemination of periodical information. To others who are favourably inclined to a community of knowledge, this journal will not fail to commend itself, as one of the most efficacious means of exposing the impositions of quackery, and imparting to honorable pretensions that kind of information which their usefulness and talents should secure.

The journal will be regularly issued on the first of December, March, June and September, annually, at the enhanced price of five dollars per volume, payable in all cases in advance. The price of subscription may be paid to the treasurer of the society, Dr. C. A. Harris, Baltimore city, or to either of the editors, or any of the publishers, collaborators or acknowledged agents of the journal. Advance payment is required by a special resolution, passed at the last meeting of the society, and is necessary to protect it from pecuniary loss, a neglect of which has involved the publishers of the first volume in unexpected embarrassment, if not in ultimate loss.

To save postage, the agents in all the large cities of the United States, and Great Britain, will be furnished with copies to supply subscribers, by such conveyance as they shall prescribe. And where it can be done, even individuals may receive their numbers by private conveyance if they choose.

With these few explanatory remarks, we commit our work to its coming destiny, after assuring the profession that no efforts will be spared to render it every way worthy of the great subject to which its pages will be exclusively devoted.

CHAPIN A. HARRIS,
AMOS WESTCOTT,
EDWIN J. DUNNING.

---

Besides the Collaborators, the following gentlemen are regularly authorized

### AGENTS OF THIS JOURNAL.

Saxton & Ketts,..........Boston.

John M'Connell, M. D.....Richmond, Va.

O. P. Laird, M. D.........Columbus, Ga.

L. S. Parmly, M. D........New Orleans.

G. McDonald, M. D ........Macon, Ga.

Drs. Goddard and Ferguson, Louisville, Ky.

James Taylor, M. D.. ....Cincinnati, Ohio.

A. Vancamp, D. D. S,......Nashville, Tenn.

A. Baldwin, D. D. S. . .....Alabama.

Nicholas Clute,...... ....Louisville,

Benj. Strickland,.........Cleveland, Ohio.

B. B. Brown, M. D.........St. Louis, Mo.

W. R. Scott, D. D. S........Raleigh, N. C.

S. M. Shepherd,............Petersburg, Va.

John A. Cleveland,.......Charleston, S. C.

Wm. F. Bason, D. D. S.,......N. Carolina.

John Lees, 23 Exchange Arcade, Manchester England.

